# A retrospective in‐depth analysis of continuous glucose monitoring datasets for patients with hepatic glycogen storage disease: Recommended outcome parameters for glucose management

**DOI:** 10.1002/jimd.12383

**Published:** 2021-05-05

**Authors:** Fabian Peeks, Irene J. Hoogeveen, R. Lude Feldbrugge, Rob Burghard, Foekje de Boer, Marieke J. Fokkert‐Wilts, Melanie M. van der Klauw, Maaike H. Oosterveer, Terry G. J. Derks

**Affiliations:** ^1^ Section of Metabolic Diseases Beatrix Children's Hospital, University Medical Center Groningen, University of Groningen Groningen The Netherlands; ^2^ enerGQ BV Groningen The Netherlands; ^3^ Department of Endocrinology University of Groningen, University Medical Center Groningen Groningen The Netherlands; ^4^ Laboratory of Pediatrics University of Groningen, University Medical Center Groningen Groningen The Netherlands

**Keywords:** continuous glucose monitoring, diabetes mellitus, empagliflozin, Fourier analysis, glycogen storage disease, person‐centered outcomes

## Abstract

Continuous glucose monitoring (CGM) systems have great potential for real‐time assessment of glycemic variation in patients with hepatic glycogen storage disease (GSD). However, detailed descriptions and in‐depth analysis of CGM data from hepatic GSD patients during interventions are scarce. This is a retrospective in‐depth analysis of CGM parameters, acquired in a continuous, real‐time fashion describing glucose management in 15 individual GSD patients. CGM subsets are obtained both in‐hospital and at home, upon nocturnal dietary intervention (n = 1), starch loads (n = 11) and treatment of GSD Ib patients with empagliflozin (n = 3). Descriptive CGM parameters, and parameters reflecting glycemic variation and glycemic control are considered useful CGM outcome parameters. Furthermore, the combination of first and second order derivatives, cumulative sum and Fourier analysis identified both subtle and sudden changes in glucose management; hence, aiding assessment of dietary and medical interventions. CGM data interpolation for nocturnal intervals reduced confounding by physical activity and diet. Based on these analyses, we conclude that in‐depth CGM analysis can be a powerful tool to assess glucose management and optimize treatment in individual hepatic GSD patients.

**Abbreviations**
#: number of measurement
*AGL*
: amylo‐1,6‐glucosidase, 4‐alpha glucanotransferase
ANC: absolute neutrophil count
AUC: area under the curve
BL: baseline
BSS: Bristol Stool Scale
CGDF: continuous gastric drip‐feeding
CGM: continuous glucose monitoring
CI: clinical intervention
cm: centimeter
CUSUM: cumulative sum
CV: coefficient of variation
d: delta
DM: diabetes mellitus
EGP: endogenous glucose production
EMPA: empagliflozin
F: female
FGM: flash glucose monitoring
G5: generation 5
G6: generation 6
*G6PC*
: glucose‐6‐phosphatase catalytic subunit
GCSF: Granulocyte Colony Stimulating Factor
GSD: glycogen storage disease
IBD: inflammatory bowel disease
kg: kilogram
M: male
Max: maximum
Min: minimum
min: minutes
P: patient
*PHKA2*
: Phosphorylase Kinase Regulatory Subunit Alpha 2
SLC37A4: Solute Carrier Family 37 Member 4
TAR: time above range
TIR: time in range
TUR: time under range
Var: variance
WMO: Law on Medical Scientific Research involving Human Beings
y: years

## 1 INTRODUCTION

Hepatic glycogen storage diseases (GSD) are rare inherited disorders of carbohydrate metabolism that can be classified according to the enzyme deficiency and organ distribution. An important biochemical hallmark of hepatic GSD is hypoglycemia, either with (GSD 0, III, IV, VI, IX, and XI) or without hyperketonemia (GSD Ia and Ib), that can lead to acute and chronic complications.^1^, ^2^ A strict diet, self‐management and self‐monitoring of glucose homeostasis are the cornerstone of treatment and aim to maintain euglycemia and prevent secondary metabolic derangement.^3^

Traditionally, metabolic control during dietary treatment is monitored based on symptoms and signs, clinical parameters (weight, height, and liver size) and circulating biomarkers, such as glucose, ketones, lactate, triglycerides, total cholesterol, and uric acid,^4^, ^5^, ^6^, ^7^, ^8^, ^9^ as well as urinary tetrasaccharide.^10^ Interpretation of these markers can be challenging due to heterogeneity between patients, and interference by medication, diet and time of sampling. Furthermore, in‐hospital or outpatient evaluations of GSD patients are expensive, invasive, time, and labor intensive and not a good reflection of everyday life at home. The recent international priority setting partnership for patients with hepatic GSD emphasized the importance of improving minimally and non‐invasive monitoring modalities of metabolic control.^11^

Continuous glucose monitoring (CGM) and flash glucose monitoring (FGM) systems are novel techniques for real‐time assessment of glucose management. Benefits of CGM include the generation of large quantities of data at home, to improve home site self‐monitoring and self‐management, and thereby patient empowerment.^12^ CGM and FGM are recommended by several (inter)national guidelines^13^, ^14^, ^15^, ^16^, ^17^; depending on the country, their use is approved and reimbursed for (subgroups of) diabetes mellitus (DM) patients. CGM is a safe and effective monitoring modality in DM patients by improving glucose management and reducing HbA1c.^18^ For DM patients experiencing a high risk of hypoglycemia, real‐time CGM can help to prevent hypoglycemia and contribute to improved quality of life.^19^ In addition, CGM has proven to be a valuable monitoring modality in dietary and medical intervention trials with DM patients.^20^

CGM is increasingly recognized as a monitoring modality for hepatic GSD patients. Previous studies have shown that CGM can uncover asymptomatic (nocturnal) hypoglycemia and can be used for monitoring of glycemic variation in individual hepatic GSD patients.^21^, ^22^, ^23^, ^24^ There is good concordance between CGM and self‐monitoring of blood glucose, which adds to the validity of the technique for home‐site monitoring.^25^ However, despite many safety issues related to fasting intolerance,^1^ unfortunately, the costs for CGM are currently not generally reimbursed for hepatic GSD patients.

In‐depth analysis of CGM datasets is rapidly emerging in the DM related literature^26^, ^27^, ^28^ and clinically meaningful CGM outcome parameters have been identified, such as time under range (TUR), time in range (TIR), and time above range (TAR).^29^ These parameters have been included as person‐centered outcomes for DM, as recently prioritized by a multidisciplinary panel of academics, healthcare professionals and patients.^30^ Furthermore, in‐depth data analysis methods such as Fourier analysis have been applied to assess glucose management in DM patients.^26^

Previous GSD guideline publications have not included recommendation for CGM outcome parameters and, there is paucity of the literature describing in‐depth CGM analysis for assessment of glucose management in individual GSD patients. We previously reported cumulative sum (CUSUM) analysis on lifelong triglyceride concentrations for assessing longitudinal metabolic control within individual GSD Ia patients.^52^ Here, we present a retrospective in‐depth analysis of CGM parameters, obtained in a continuous, real‐time fashion during different dietary and medical interventions, assessing glucose management in 15 individual GSD patients.

## 2 METHODS

### 2.1 Ethical approval

The Medical Ethical Committee of the University Medical Center Groningen (UMCG) confirmed that the Law on Medical Scientific Research involving Human Beings (WMO) did not apply to the current study (MEC 2019‐119).

### 2.2 Study design

This was a monocenter, retrospective, observational study of CGM data obtained in a real‐time fashion in three subsets of hepatic GSD patients who underwent dietary and medical interventions at the UMCG, Groningen, the Netherlands.

### 2.3 Subjects

Hepatic GSD patients at the UMCG received CGM to support GSD clinical and home evaluations and as safety measure during in‐hospital dietary or medical interventions. The first CGM dataset was obtained from a single GSD Ia patient in whom multiple dietary interventions were performed, including exchanging nocturnal continuous gastric drip feeding (CGDF) for a nocturnal uncooked cornstarch (UCCS) regimen, both in‐hospital and at home. The second CGM dataset was obtained from 11 GSD patients during in‐hospital starch load tests that were part of the GLYDE trial (NCT02318966). In brief, this was a prospective, randomized, double‐blind, crossover trial of Glycosade vs UCCS in which the first part consisted of two (blinded) 12‐hour starch load tests. For the current study, we compared the CGM data obtained from UMCG patients during both starch loads; results on the GLYDE trial will be published elsewhere. The third CGM dataset was obtained from three GSD Ib patients during the in‐hospital initiation and at home follow‐up of the off‐label treatment with empagliflozin for neutropenia and neutrophil dysfunction associated symptoms and signs.^31^

### 2.4 Continuous glucose monitoring systems

The Dexcom CGM Systems are mostly applied in DM and they are approved for children of 2 years and older on the abdomen and lower back. The first and the third CGM subsets were obtained using Dexcom G6 (Dexcom, San Diego, California). The second CGM subset was obtained using Dexcom G4. Whereas both systems allow real‐time CGM measurements, the Dexcom G6 additionally supports real‐time online data sharing. Both Dexcom G4 and G6 have a relatively high accuracy of measurements in hypoglycemic range and sensitivity for detecting hypoglycemia in DM patients.^32^, ^33^ Herbert and co‐workers described a strong correlation between Dexcom G4 CGM values and standard capillary blood glucose measurements in patients with hepatic GSD, also in the range <3.9 mmol/L.^25^

The CGM device consists of a wireless receiver, a transmitter and a sensor. The sensor is inserted in the subcutaneous tissue in the interstitial space. The sensor coated with glucose oxidase reacts with glucose, producing an electrical current every 5 minutes (288 measurements per day). The blood glucose concentration is derived from the subcutaneous glucose concentration with computer‐driven algorithms, where after the measurement is transmitted to the wireless receiver. For Dexcom G4, the sensor requires calibration twice a day by capillary glucose measurement, whereas Dexcom G6 is factory calibrated.

### 2.5 Outcome parameters and data analysis

From the medical files, additional baseline and demographic patient information was collected on GSD type, mutation, gender, age, type of dietary treatment before and after intervention, medication, and parameters of metabolic control.

The raw data files were retrieved from the Dexcom CLARITY Clinical Portal (https://clarity.dexcom.eu/professional/patients) and stored anonymously as CSV‐files before analysis.^34^, ^35^ The Dexcom CGM System is validated for glucose concentrations above 2.2 mmol/L (>40 mg/dL). If the CGM sensor indicated a low value, the lowest possible CGM value of 2.2 mmol/L was used, since omitting these values would bias the descriptive statistics.

Statistical analysis was performed using IBM SPSS Statistics 23. In the second CGM dataset, group differences were calculated by paired *t*‐test. *P*‐values lower than .05 were considered significant (two‐tailed).

Primary outcomes from the CGM dataset included descriptive outcomes (median, minimum, maximum, range), outcomes of glycemic variation (SD, variance, coefficient of variation [CV, calculated as SD divided by the mean]) and outcomes of glycemic control such as TUR as glucose ≥3.0 mmol/L and < 3.9 mmol/L (ie, level 1 hypoglycemia), as glucose <3.0 mmol/L (ie, level 2 hypoglycemia) and as level 3 hypoglycemia (ie, a severe event characterized by altered mental and/or physical status requiring assistance), TIR as either more physiological CGM values ≥3.9 and ≤7.8 mmol/L or ≥ 3.9 and ≤10.0 mmol/L, and TAR as either CGM values >7.8 mmol/L or CGM values >10.0 mmol/L, defined according to the American Diabetes Association 2020.^15^ We assumed that diurnal variations of the actual diet and physical activity are confounding factors of glucose homeostasis. To correct for these factors, the CGM values between 1:00 and 5:00 am. were analyzed separately for subsets I and III.

Secondary outcomes included the following:

1. CGM glucose concentrations directly derived from the Dexcom CLARITY Clinical Portal.
2. Descriptive statistics of glucose concentrations of the interval between 1:00 and 5:00 am.
3. The first order derivative (change over time) calculated as $\text{glucos}e^{\prime }=\frac{\text{dglucose}}{\mathrm{dt}}$.
4. The second order derivative (speed of change over time) calculated as $\text{glucos}e^{\prime \prime }=\frac{d^{2}\text{glucose}}{dt^{2}}$.
5. To display subtle variations for repeated measurements, CUSUM graphs were constructed of the time interval 1:00‐5:00 pm. based on two methodologies. Method A has been described previously^52^, and the CUSUM for serial measurements was calculated as ${\text{CUSUM}}_{t}=\sum_{i=1}^{t}{\text{glucose}}_{i}-{\text{mean glucose}}_{i}$. Method B corrected for first and second order derivatives, and the CUSUM was calculated as ${\text{CUSUM}}_{t}=\sum_{i=1}^{t}\text{glucose}\text{\_}{\text{calculated}}_{i}-{\text{glucose}}_{i},$ where glucose_calculated was determined using a hypercube regression analysis based on the average numerical dataset per individual patient that correlated absolute glucose values to first and second order derivatives.
6. Fourier analysis is a technique to visualize complex (homeostasis) patterns of CGM data and was performed as described previously^27^ by mathematically transforming the CGM data with a Fast Fourier Transformation (FFT) and expressing the data as one or more sinusoidal curves. The amplitude is calculated as a representation of the phase of the CGM signal as $\text{Amplitude}=2*\mathrm{abs}\left(\frac{\mathrm{FFT}\text{\_}\text{Result}}{n}\right)$. From the mathematical transformation, a spectrogram is constructed that identifies three outcome parameters that have implications for glucose management: the frequency (the number of cycles per night), the number of frequencies (a glucose pattern can consist of one frequency or multiple patterns) and the amplitude of each identified frequencies. Good glucose management is characterized by a low frequency, a low number of frequencies and a small amplitude.

## 3 RESULTS

Table 1 presents the general characteristics of the 15 included hepatic GSD patients (3 GSD Ia, 3 GSD Ib, 4 GSD IIIa, 3 GSD IXα, 2 GSD IX). We analyzed CGM data from 11 male and four female patients, ages ranged from 2 to 22 years.

**TABLE 1:** General characteristics of the hepatic GSD patients from the three CGM subsets

Subset	Patient	Age (y)^a^	Sex	GSD type	Gene	Mutation 1	Mutation 2
I	P‐I‐1	9	M	Ia	*G6PC*	c.888G>T	c.888G>T
II	P‐II‐1	22	F	Ia	*G6PC*	c.79delC	c.79delC
II	P‐II‐2	8	M	IIIa	*AGL*	c.3911del	c.3911del
II	P‐II‐3	7	M	IIIa	*AGL*	c.3911del	c.3911del
II	P‐II‐4	2	M	IXα	*PHKA2*	c.3614C>T	‐
II	P‐II‐5	12	M	IXα	*PHKA2*	c.3614C>T	‐
II	P‐II‐6	13	M	IXα	*PHKA2*	c.601C>T	‐
II	P‐II‐7	12	F	IIIa	*AGL*	c.1020del	c.1020del
II	P‐II‐8	15	F	Ia	*G6PC*	c.79delC	c.209G>A
II	P‐II‐9	2	M	IX	*‐*	Unknown^b^	Unknown^b^
II	P‐II‐10	6	M	IX	*‐*	Unknown^b^	Unknown^b^
II	P‐II‐11	10	M	IIIa	*AGL*	c.1222C>T	c.2120_2121delAA
III	P‐III‐1	6	M	Ib	*SLC37A4*	c.1042_1043delCT	c.1042_1043delCT
III	P‐III‐2	2	F	Ib	*SLC37A4*	c.1042_1043delCT	c.899G>A
III	P‐III‐3	11	M	Ib	*SLC37A4*	c.365G>A	c.365G>A

Abbreviations: *AGL*, amylo‐1,6‐glucosidase, 4‐alpha glucanotransferase; CI, clinical intervention; EMPA, empagliflozin; *G6PC*, glucose‐6‐phosphatase catalytic subunit; GSD, glycogen storage disease; P, patient; *PHKA2*, Phosphorylase Kinase Regulatory Subunit Alpha 2; *SLC37A4*, Solute Carrier Family 37 Member 4; y, years.

^a^ Age at start of study.

^b^ Diagnosis based on deficient phosphorylase kinase activity.

### 3.1 CGM subset I: nocturnal dietary interventions

This nine‐year‐old male GSD Ia patient was referred to our clinic for GSD evaluation. He suffered from recurrent hypoglycemia on his former dietary regimen and UCCS intolerability was reported, leading to diarrhea. He was treated with daytime frequent feeds with either a meal or nasogastric tube feed based on short‐acting carbohydrates (*Frebini* original fiber). During daytime, he used 25 g of UCCS every 3 hours. At night, he received CGDF (*Nutridrink Juicy Style* of 47 kcal/hour, 10.4 g/hour or 4.6 mg/kg/minutes of carbohydrates), whereas without GSD, his calculated, predicted endogenous glucose production (EGP) would approximate 4.2 mg/kg/minutes and 3.7 mg/kg/minutes at his current weight of 37.4 kg^36^ and age,^37^ respectively. Metabolic control was summarized by his biometry (BMI: +3.8 SDs; Weight‐for‐height + 5.2 SDs), high serum triglyceride (9.4 mmol/L) and total cholesterol (6.9 mmol/L) concentrations, and severe hepatomegaly assessed by abdominal ultrasound (26 cm maximal craniocaudal distance; normal range 9 to 11 years: 7.5 to13.5 cm^38^). After several attempts to monitor and adjust dietary treatment at home including reducing nocturnal CGDF, it was decided to perform an in‐patient evaluation because of persisting hypoglycemias and excessive weight gain.

**FIGURE 1:**
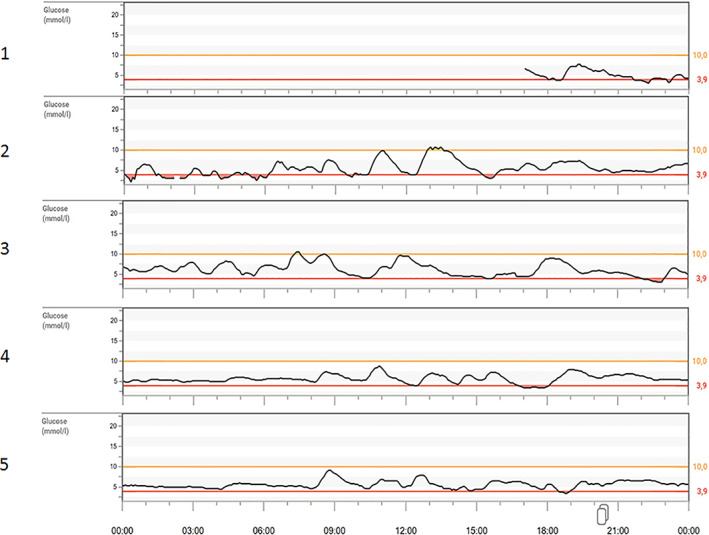
CGM subset I from the Dexcom Clarity Clinical Portal of the in‐hospital evaluation of P‐I‐1. The 5 days of in‐patient evaluation of P‐I‐1. Day 1 the current treatment of *Nutridrink Juicy Style* was evaluated. Days 2 and 3 the Maltodextrin (metaX—Institut für Diätetik GmbH) intervention is evaluated. Days 4 and 5 the UCCS intervention was evaluated. P, patient; UCCS, uncooked cornstarch

Figure 1 illustrates the Dexcom graphs, directly exported from the Dexcom CLARITY Clinical Portal, presenting the CGM data obtained during the in‐hospital evaluation. Table 2 presents the CGM data analysis per intervention. After baseline assessment of the existing dietary regimen on the first day (and night) of in‐hospital stay, caloric intake was restricted during the second and third night by changing to a MaltoCal 6 solution (metaX—Institut für Diätetik GmbH; 34.6 kcal/hour, 8.6 g/hour, 3.9 mg/kg/minutes of carbohydrates). Despite the reduction in carbohydrate intake after introduction of MaltoCal 6, a strong decrease of TUR could already be observed. During the fourth night, instead of nocturnal CGDF, an UCCS regimen was introduced of 45 g at 20:00, 0:00 and 4:00 (40 kcal/hour, 9.9 g/h, 4.4 mg/kg/minutes). The nocturnal UCCS regimen (days 4 and 5) stabilized CGM profiles and further reduced the amount of TUR. The variables of glycemic variation (variance, SD, and CV) also strongly decreased, presumably indicating episodes of hyperinsulinism. After a week at home, the nocturnal UCCS doses were lowered to 40 g (35 kcal/hour, 8.8 g carbohydrates/hour, 3.9 mg glucose/minutes/kg).

**TABLE 2:** CGM subset I of nocturnal continuous gastric drip feeding (1, 2) and uncooked cornstarch (3, 4) interventions of P‐I‐1

Interventions	#	Days/nights	Median (mmol/L)	Min (mmol/L)	Max (mmol/L)	SD (mmol/L)	Var (mmol^2^/L^2^)	CV (%)	95% CI (mmol/L)	TUR <3.0 (%)	TUR 3.0‐3.9 (%)	TIR 3.9‐7.8 (%)	TIR 3.9‐10.0 (%)	TAR >7.8 (%)	TAR >10.0 (%)
**1. Nutridrink Juicy Style**															
Total	1197	4	4.5	2.2	7.6	1.1	1.2	24.4	2.3‐6.7	0.1	17.4	73.7	73.7	0.0	0.0
01:00–5:00	192	4	4.3	2.3	6.0	0.9	0.8	21.4	2.4‐6.1	10.4	18.8	70.8	70.8	0.0	0.0
**2. Maltocal 6**															
Total	577	2	5.4	2.2	10.5	1.7	2.9	29.5	2.4‐9.2	0.0	9.7	77.3	88.4	12.8	1.7
01:00–5:00	94	2	5.5	3.1	8.1	1.6	2.5	29.4	2.2‐8.5	0.0	24.5	70.2	75.5	5.3	0.0
3. UCCS*															
Total	1890	7	5.8	2.9	9.7	1.1	1.2	18.2	3.8‐8.1	0.0	2.4	92.1	97.5	5.3	0.0
01:00–5:00	335	7	5.7	4.5	7.4	0.6	0.4	11.1	4.5‐7.1	0.0	0.0	100.0	100.0	0.0	0.0
4. UCCS follow‐up**															
Total	3882	14	5.8	2.3	10.8	1.4	2.1	23.4	3.3‐9.0	0.0	2.3	81.9	97.1	15.5	0.3
01:00–5:00	672	14	5.0	2.5	8.3	0.6	0.4	12.6	3.8‐6.3	0.3	2.1	97.5	97.6	0.1	0.0

*Note*: The first intervention included baseline measurements. * UCCS was given at 20:00, 0:00 and 4:00. ** Follow‐up was performed 9 months after the initial visit and the CGM data was collected in an outpatient setting.

Abbreviations: CI, confidence interval; CV, coefficient of variation; Max, maximum; Min, minimum; TAR, time above range; TIR, time in range; TUR, time under range; UCCS, uncooked cornstarch; Var, variance; %, percent; #, number of measurements; <, below; >, above.

After 9 months of follow‐up, during an outpatient visit, improvements were observed in biometry (BMI: +2.4 SDs; weight‐for‐height + 3.7 SDs), hyperlipidemia (serum triglyceride 5.4 mmol/L; total cholesterol 6.0 mmol/L) and hepatomegaly (19 cm craniocaudal distance). Evaluation of 13 days of home‐site CGM data showed a low percentage of time spent in hypoglycemia, with limited variance and SD of the data (Table 2).

### 3.2 CGM subset II: starch loads

In Table 3, the grouped results of the starch load tests with either UCCS or Glycosade are presented. In general, the Glycosade starch loads showed lower maximum glucose concentrations and which occurred later with a smaller range as compared to the UCCS starch loads. Additionally, the initial, increasing slope of the graph from the start to the maximum glucose concentration was less steep with the Glycosade starch load suggesting a more gradual intestinal glucose absorption. There is a trend toward smaller glycemic variation in response to the Glycosade starch load. Table 4 presents the CGM data from individual GSD patients, in response to different products, which may depend on different factors, such as GSD subtype, age, and gender.

**TABLE 3:** CGM subset II of uncooked cornstarch and glycosade starch loads per group

Starch load	Median (mmol/L)	Min (mmol/L)	Max (mmol/L)	Range (mmol/L)	SD (mmol/L)	Var (mmol^2^/L^2^)	CV (%)	Initial glucose value (mmol/L)	Time to max (min)	End time (min)	Slope 0 to max (mmol/L/h)	Slope max to end (mmol/L/h)	AUC (mmol*h)
UCCS	4.9	3.3	8.8	5.5	1.3	1.8	25.1	4.6	69	576	4.8	−0.6	49
Glycosade	4.9	3.7	7.9	4.3	1.0	1.1	19.9	5.1	80	584	3.0	−0.5	51
P‐value	0.859	0.321	0.038	0.036	0.107	0.190	0.177	0.298	0.369	0.757	0.008	0.099	0.895

*Note*: In the crossover design, patients were randomized to either UCCS or Glycosade starch loads first. * A *P*‐value <.05 is considered significant. Values per starch load are calculated as means of all patients. The Glycosade intervention of P‐II‐2 and P‐II‐3 was excluded in group analyses due to incomplete CGM data.

Abbreviations: AUC, area under the curve; cm, centimeter; CV, coefficient of variation; F, female; kg, kilogram; Max, maximum; min, minutes; Min, minimum; M, male; P, patient; UCCS, uncooked cornstarch; Var, variance; y, years; #, number of measurements.

**TABLE 4:** CGM subset II of uncooked cornstarch and glycosade starch loads per individual patient

P	Age (y)	Sex	GSD Type	Height (cm)	Weight (kg)	Starch load	#	Median (mmol/L)	Min‐Max (Range) (mmol/L)	SD (mmol/L)	Var (mmol^2^/L^2)^	CV (%)	Initial value (mmol/L)	Time to max (min)	End time (min)	End value (mmol/L	Slope 0‐max^a^	Slope max‐end^a^	AUC (mmol*h)
II‐1	22	F	Ia	149	29.4	UCCS	96	5.0	3.0‐8.4 (5.4)	1.5	2.3	29.4	4.0	185	475	3.0	1.4	−1.1	41
						Glycosade	96	5.1	3.1‐7.3 (4.2)	1.1	1.3	21.2	4.1	215	475	3.1	0.9	−1.0	41
II‐2	8	M	IIIa	132	17.2	UCCS	93	4.9	3.6‐8.7 (5.2)	1.4	1.8	26.4	4.7	40	460	3.6	6.0	−0.7	41
						Glycosade	73	^b^	^b^	^b^	^b^	^b^	^b^	^b^	550	4.3	^b^	^b^	^b^
II‐3	7	M	IIIa	132	16.6	UCCS	108	4.9	3.2‐7.9 (4.7)	1.1	1.1	22.0	4.9	70	535	3.2	2.5	−0.6	45
						Glycosade	61	^b^	^b^	^b^	^b^	^b^	^b^	^b^	475	4.7	^b^	^b^	^b^
II‐4	2	M	IX	82	17.4	UCCS	98	5.6	4.1‐9.3 (5.3)	1.0	1.1	17.5	5.1	100	485	5.2	2.6	−0.6	47
						Glycosade	108	4.7	3.3‐7.9 (4.6)	0.9	0.9	18.4	4.9	75	535	4.9	2.5	−0.4	44
II‐5	12	M	IX	136	20.4	UCCS	146	4.3	3.2‐8.1 (4.9)	1.0	0.9	21.7	5.0	55	725	4.8	3.4	−0.3	55
						Glycosade	146	3.7	3.2‐7.1 (3.9)	0.9	0.8	22.5	4.8	40	725	4.8	3.4	−0.2	49
II‐6	13	M	IX	154	14.8	UCCS	146	4.7	3.3‐8.6 (5.3)	1.0	1.0	20.4	3.3	40	725	4.3	7.9	−0.4	59
						Glycosade	144	5.2	3.7‐7.5 (3.8)	0.7	0.5	13.2	3.7	40	715	4.9	5.7	−0.2	63
II‐7	12	F	IIIa	136	81.0	UCCS	143	4.4	2.5‐7.4 (4.9)	1.1	1.2	24.4	4.9	35	710	2.5	4.4	−0.4	54
						Glycosade	108	5.2	3.6‐6.8 (3.2)	0.9	0.8	18.4	6.6	90	535	3.7	0.1	−0.4	44
II‐8	15	F	Ia	159	25.3	UCCS	120	5.4	3.4‐6.2 (2.8)	0.6	0.4	11.5	3.4	100	595	3.7	1.7	−0.3	52
						Glycosade	122	5.8	4.7‐7.4 (2.7)	0.7	0.5	12.1	5.8	115	605	5.0	0.8	−0.3	59
II‐9	2	M	IX	86	13.4	UCCS	86	3.9	2.0‐12.1 (10.1)	2.5	6.2	56.8	5.5	40	425	2.5	9.9	−1.5	31
						Glycosade	98	4.7	3.9‐8.7 (4.8)	1.3	1.8	25.0	4.1	45	485	4.2	6.1	−0.6	42
II‐10	6	M	IX	125	24.4	UCCS	122	5.0	4.1‐10.2 (6.1)	1.4	2.0	25.5	5.2	45	605	4.9	6.6	−0.6	55
						Glycosade	122	3.9	3.2‐8.8 (5.7)	1.2	1.5	27.3	4.4	60	605	3.8	4.4	−0.6	44
II‐11	10	M	IIIa	142	43.8	UCCS	120	5.5	4.3‐9.9 (5.6)	1.2	1.4	20.3	4.9	50	595	4.3	5.9	−0.6	59
						Glycosade	145	5.9	4.2‐9.7 (5.5)	1.4	2.1	21.5	7.8	40	725	4.9	2.8	−0.4	78

*Note*: In the crossover design, patients were randomized to either receive the UCCS or Glycosade intervention first.

Abbreviations: AUC, area under the curve; cm, centimeter; CV, coefficient of variation; F, female; kg, kilogram; Max, maximum; min, minutes; Min, minimum; M, male; P, patient; UCCS, uncooked cornstarch; Var, variance; y, years; #, number of measurement.

^a^ Unit is mmol/L/h.

^b^ Start of CGM measurements after peak of glucose‐measurement.

### 3.3 CGM subset III: empagliflozin in GSD Ib patients

**FIGURE 2:**
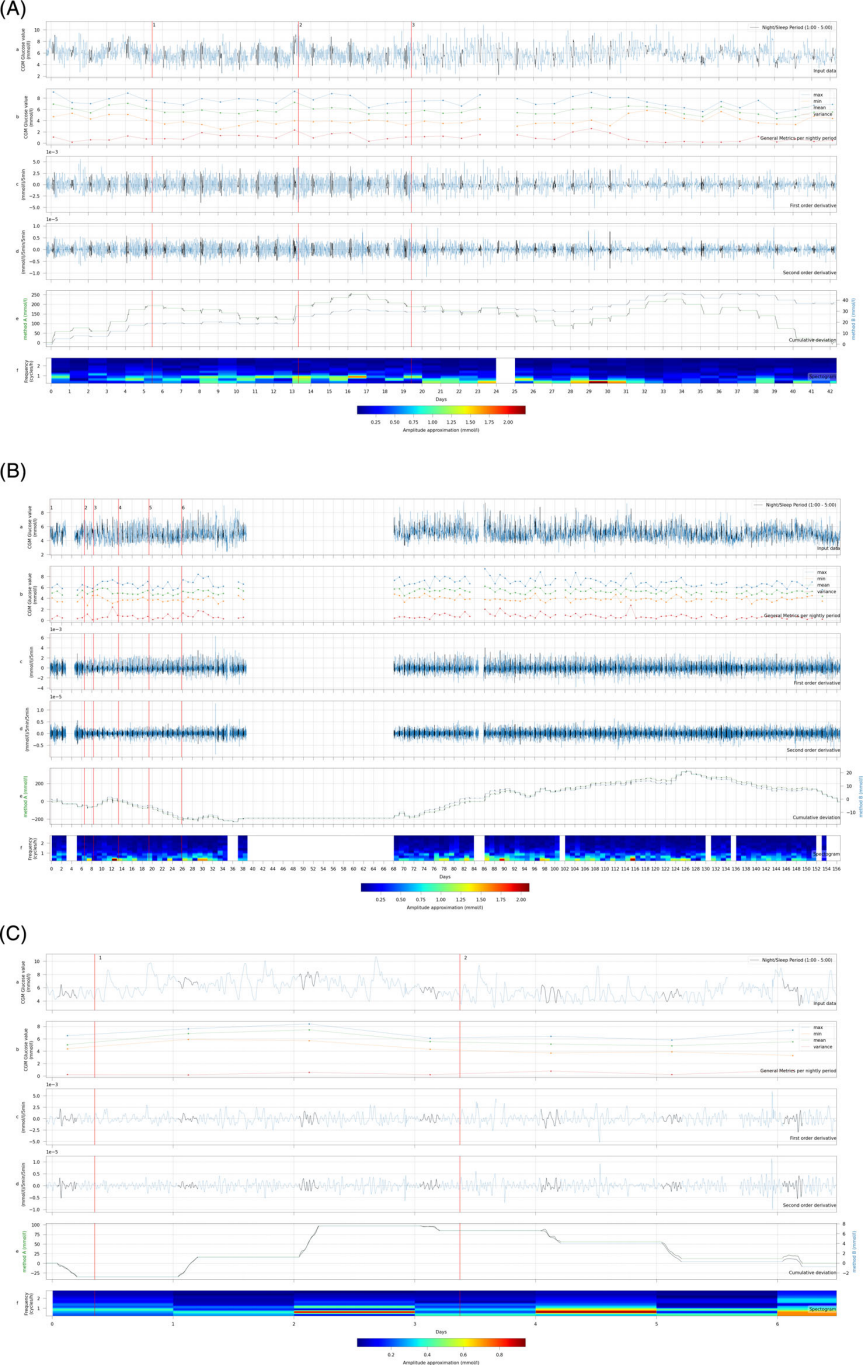
In‐depth data analysis of CGM subset III of GSD Ib patients treated with empagliflozin. A. P‐III‐1. B. P‐III‐2. C. P‐III‐3. P‐III‐1:1 = Empagliflozin 5 mg 1dd1 2 = Empagliflozin 5 mg 2dd; 3 = UCCS; P‐III‐2:1 = Empagliflozin 5 mg 1dd; 2 = UCCS 25 g 6dd; 3 = Empagliflozin 7.5 mg 1dd; 4 = Empagliflozin 5 mg 1dd and 2.5 mg 1dd; 5 = Empagliflozin 5 mg 2dd; 6 = Empagliflozin 5 mg 2dd (second dose giver at 20:00 instead of 16:00). P‐III‐3:1 = Empagliflozin 10 mg 2dd; 2 = Empagliflozin 15 mg 1dd and 10 mg 1dd. In blue the complete data is described, the data in black represent the interval between 1:00‐5:00 am. a. CGM concentrations; b. Descriptive data (mean, maximum, minimum, variation) between 1:00‐5:00 am; c. First order derivative of CGM concentrations; d. Second order derivate of CGM concentrations; e. Cumulative sum analysis method A (in blue, left axis) and cumulative sum analysis method B (in green, right axis); f. Fourier analysis spectrogram of CGM profile between 1:00‐5:00 am. The y‐axis displays the frequency of the sinusoidal CGM pattern in cycles per hour. The color displays the amplitude of the frequency (waterfall plot JOT color scheme). CGM, continuous glucose monitoring; dd, times per day; GSD, glycogen storage disease; P, patient; UCCS, uncooked cornstarch

Figure 2 illustrates the in‐depth CGM data analysis of three GSD Ib patients treated with empagliflozin. In these patients, CGM is mainly used for safety reasons to detect possible hypoglycemia as a potential side effect of empagliflozin‐induced glucosuria. The case history of P‐III‐1 was reported elsewhere.^31^ Table 5 presents details of descriptive data per intervention. Although glucose concentrations remained <4.0 mmol/L for a longer period of time in all three patients after initiation of empagliflozin, no symptoms of hypoglycemia are reported.

**TABLE 5:** CGM subset III of GSD Ib patients treated with empagliflozin

P	Int	Med	Dose	Days^a^	n	Median^a^	Min^a^	Max^a^	SD ^a^	Var	CV (%)	95% CI	TUR <3.0^a^	TUR 3.0–3.9^a^	TIR 3.9–7.8^a^	TIR 3.9–10.0^a^	TAR >7.8^a^	TAR >10.0^a^
III‐1	BL	None	‐	0‐5.5	1602	6.2	2.7	9.5	1.1	1.2	17.5	4.0‐8.4	0.1	1.4	90.9	98.5	7.6	0.0
	1	Empa	5 mg	5.5‐13.5	2212	5.6	2.5	11	1.1	1.3	20.2	3.4‐7.9	0.4	4.2	92.1	95.2	3.3	0.2
	2	Empa	5 mg 2dd	13.5‐19.5	1694	5.7	2.4	9.8	1.1	1.3	20.2	3.4‐8.0	0.4	4.4	92.0	95.2	3.2	0.0
	3	UCCS	38 g 7dd	19.5‐42.5	6495	5.8	2.2	10.9	1.2	1.4	20.2	3.4‐8.1	0.4	3.1	91.6	96.2	4.8	0.2
	4^c^	Empa	7.5 mg + 5 mg	42.5‐109.5	11 310	5.5	2.4	10.6	1.0	1.0	17.7	3.6‐7.5	0.2	3.4	94.8	96.3	1.6	0.1
	5^c^	Empa	7.5 mg + 7.5 mg	109.5‐178	6513	5.6	2.2	10.5	1.1	1.2	19.4	3.4‐78	0.7	3.5	92.6	95.7	3.2	0.0
	6^c^	Empa	10 mg + 10 mg	178‐238	3675	5.4	2.3	11.7	1.0	1.0	18.6	3.4‐7.4	0.4	5.6	93.1	93.9	1.0	0.1
III‐2	1	Empa	5 mg	0–7	1502	4.9	2.7	7.0	0.8	0.6	15.3	3.4‐6.4	0.3	0.3	7.8	91.9	0.0	0.0
	2	UCCS	25 g 6dd	7–9	546	4.9	2.7	7.0	0.9	0.7	17.5	3.2‐6.6	0.7	0.7	11.5	87.7	0.0	0.0
	3	Empa	7.5 mg	9‐14	1447	4.7	2.9	7.6	0.9	0.8	19.4	2.9‐6.6	0.1	0.1	17.7	82.2	0.0	0.0
	4	Empa	5 mg + 2.5 mg	14‐19	1731	5.0	3.1	7.3	0.9	0.8	18.0	3.2‐6.8	0.0	0.0	9.8	90.2	0.0	0.0
	5	Empa	5 mg 2dd	19‐25	1851	4.7	2.9	7.3	0.8	0.6	15.9	3.2‐6.2	0.1	0.1	9.7	90.2	0.0	0.0
	6	Empa	5 mg 2dd^b^	25‐157	28 271	5.1	2.3	9.6	0.9	0.7	16.9	3.4‐6.8	0.2	0.2	4.1	95.1	0.7	0.0
III‐3	BL	None	‐	0‐0.5	117	5.1	4.2	6.7	0.6	0.3	11.0	4.0‐6.3	0.0	0.0	100.0	100.0	0.0	0.0
	1	Empa	10 mg 2dd	0.5‐3.5	872	6.5	3.9	10.7	1.2	1.5	19.2	4.0‐9.0	0.0	0.0	88.4	99.2	11.6	0.8
	2	Empa	15 mg + 10 mg	3.5‐6.5	861	5.3	3.0	9.4	1.1	1.3	21.7	3.0‐7.5	0.0	7.4	89.2	92.6	4.1	0.0

*Note*: CGM data measurements of entire day. P‐III‐1 was discharged from hospital at day 67. P‐III‐2 was discharged at day 33. P‐III‐3 was discharged after removal of the CGM sensor.

Abbreviations: BL, baseline; CI, confidence interval; CV, coefficient of variation; dd, times per day; Empa, Empagliflozin; g, gram; Max, maximum; Med, medication; Min, minimum; mg, milligram; n, number of measurements; Int, intervention; TUR, time under range; Var, variance; %, percent; <, below.

^a^ Unit = mmol/L.

^b^ Second dose of Empagliflozin given at 20:00 instead of 16:00.

^c^ Part of the CGM data was acquired in a home‐setting.

For P‐III‐1, the CGM concentrations during the day were more stable after start of empagliflozin (intervention 1), but variability initially increased during the night as indicated by the higher first and second order derivative and a decrease in the CUSUM (Figure 2A). Furthermore, the Fourier analysis showed an increase in the number of frequencies, indicating a less stable glucose pattern. Fecal calprotectin increased from 190 (day 4) to 750 mg/kg (day 10) upon empagliflozin treatment reflecting improved neutrophil function. After a few days of treatment, CGM values increased after which the empagliflozin dose was doubled. Paradoxically, the CUSUM increased steadily after the increase in dose in parallel to decreasing fecal calprotectin (255 mg/kg; day 19) and clinically, more solid and less frequent stools were observed. Additionally, Fourier analysis showed a decrease in the number of frequencies, indicating a more stable glucose pattern. Together, these results suggest improved gastro‐intestinal absorption as a result of empagliflozin treatment. After the initiation nocturnal UCCS treatment instead of CGDF (intervention 3), there was less variation in absolute glucose concentrations and the first and second order derivatives were smaller. Additionally, the Fourier analysis illustrated a strong and sudden decrease in frequency (number of cycles per night) and the amplitude. In parallel, fecal calprotectin values normalized (55 mg/kg on day 33). After lowering the dose of granulocyte colony‐stimulating factor (day 33), the variation of CGM data decreased even further.

For P‐III‐2, a week after initiation of empagliflozin treatment, the CGM values and defecation pattern improved. UCCS was introduced (intervention 2), reducing the amount of CGDF during the day and night. Afterwards, the CGM values increased further and the first and second order derivatives decreased (Figure 2B). In parallel, the Fourier analysis showed that the frequency of the CGM data decreased, indicating less glycemic variability during the night. The stepwise increase in empagliflozin dose (interventions 3, 4, and 5) was paralleled by higher nocturnal CGM values. Interestingly, Fourier analysis identified several clinically relevant moments. At day 12, the patient experienced a relative hypoglycemia (3.4 mmol/L) accompanied by diarrhea after which an additional feed was given. Fourier analysis registered the event with an increased amplitude. On days 28‐30, the parents of the patient tried different new snacks, and glucose concentrations were relatively low (3.7 mmol/L) with multiple frequencies indicating less stable glucose homeostasis. The Fourier analysis revealed an increase of the number of frequencies and a higher amplitude. From the additional follow‐up data from home, the CGM values remained stable and the CUSUM increased further.

For P‐III‐3, empagliflozin was titrated over a period of 7 days. After initiation of empagliflozin (intervention 1), the absolute CGM values increased, as also illustrated by a rise in the CUSUM. After the increase of the empagliflozin dose (intervention 2), the CGM values did not change significantly and fecal calprotectin levels remained low (<40 mg/kg feces).

## 4 DISCUSSION

This is the first study that employs in‐depth CGM dataset analysis to evaluate these outcome parameters for glucose management in hepatic GSD patients during different dietary and medical interventions. The combination of first and second order derivatives, CUSUM analysis and Fourier analysis (the frequency, number of frequencies, and amplitude of each frequency) identified both subtle and sudden changes in glucose homeostasis that cannot be easily detected alone when focusing on pre‐prandial glucose concentrations or measuring at symptomatic hypoglycemias. Additionally, data interpolation of nocturnal intervals provides a useful strategy to limit confounding of glucose homeostasis by physical activity and diet.

The first case description illustrates how CGM data can be interpreted in a case‐oriented fashion together with clinical domain knowledge, such as traditional parameters of metabolic control. The nocturnal dietary intervention with UCCS reduced time spent in hypoglycemia and glycemic variation for this GSD Ia patient. In DM types I and II, these parameters associate with risk of severe hypoglycaemia.^39^, ^40^ Although glycemic variation appears to be smaller in hepatic GSD patients than in DM type 1 patients, fasting intolerance and risk of hypoglycemia are nevertheless strong arguments to limit glycemic variation by dietary interventions.^41^

The second CGM subset demonstrates that Glycosade may lead to less glycemic variation compared to UCCS (Table 3). However, as there is large heterogeneity in ages and GSD subtypes of these individual patients, the cumulative data should be carefully evaluated, emphasizing the importance to analyze the data in a case‐oriented, personalized manner (Table 4). This is also displayed in the third CGM subset, in which, after an initial period of lower CGM concentrations, glycemic variation decreases with possibly better uptake of dietary macronutrients and UCCS by improved gastro‐intestinal absorption after empagliflozin.

Several limitations of this study need to be addressed. First, there is as of yet no reference CGM data for GSD patients and no comparison with healthy subjects has been made. Therefore, in clinical practice and in our study, patients serve as their own control. Prospective and validation studies are warranted to determine reference values for CGM outcome parameters and to correct for intra‐patient comparisons. Second, the retrospective study design introduced information and selection bias. Third, it is currently unknown how many measurements are minimally required to obtain a reliable profile of glucose management in hepatic GSD patients. In DM patients, it has been recommended to collect 14‐day CGM data to adequately predict glycemic variability over a three‐month period.^42^, ^43^ Advanced CGM systems may allow for glucose concentration measurements with higher frequency (ie, one reported measurement per minute), thereby accelerating adequate prediction of glycemic variability. Fourth, it should be mentioned that glucose management as assessed with CGM should be balanced against psychosocial well‐being and quality of life.^44^, ^45^, ^46^ Fifth, there are several limitations to the use of CGM technology and the interpretation of interstitial fluid glucose levels. There is a delay of 10 to 15 minutes in both diffusion from capillary blood into interstitial fluid and the sensor as well as sensor signal processing time.^47^ The combination of measurement errors and physiological differences between these compartments affects CGM accuracy. Finally, the amount of subcutaneous fat impacts the accuracy of CGM, which is especially important in infants and patients with increased BMI. Therefore, to limit variability of CGM sensor location on data interpretation we advise to place the CGM sensor in the same location on either upper arm or abdomen in each individual patient.

In DM, the potential of machine learning approaches for detection of glucose abnormalities, prediction of glucose concentrations, clinical decision making, and patient education is increasingly recognized.^48^ Real‐time machine learning of CGM data in DM accurately predicts glycemic fluctuations 1 hour in advance.^49^ In theory, accumulation of big personal health data from physiological monitoring wearables offers opportunities for augmented intelligence and machine learning to improve patient self‐management and remote monitoring by healthcare providers.^48^ Supervised CGM machine learning algorithms with CUSUM and Fourier data analysis may allow for prediction of glycemic variation and outcomes in GSD patients, detecting conditions such as dietary undertreatment, overtreatment, nocturnal hypoglycemias and inflammatory bowel disease (IBD) exacerbations in GSD Ib patients. Current limiting factors include the lack of interoperability and data exchange between CGM software, hospital electronic health records, and personal digital health environment systems, to integration of multiple datasets, such as CGM data, dietary information, symptoms and signs, clinical parameters (weight, height, and liver size) and biomarkers.

The in‐depth analysis of CGM data described in this study is crucial for future application of CGM systems as clinical trials with novel medical (gene therapy and mRNA therapy) and dietary treatments for GSD patients are emerging rapidly. For mRNA therapy in hepatic inborn errors of metabolism, the route of administration is by multiple intravenous injections.^50^ For adeno‐associated virus vector‐mediated gene therapy, the duration of efficacy after a one‐time infusion is currently under clinical investigation. Corticosteroids are often used to manage presumed capsid‐triggered immune response but are also known to interfere with glucose homeostasis. To date, blood glucose levels (NCT03665636), invasive, in vivo starch load tests (NCT02318966) and controlled fasting challenges (NCT03517085) are considered relevant outcome parameters in clinical trials for GSD patients. The insights from the current study emphasize that CGM can be used as minimally invasive outcome parameter to assess safety and efficacy during intervention trials in GSD patients.

In conclusion, this study demonstrates the potential of in‐depth CGM data analysis for hepatic GSD patients during both dietary and medical interventions. Most CGM outcome parameters can be easily calculated from the raw export data from the CGM software system and can be used by any healthcare professional. CGM should be co‐interpreted with clinical domain knowledge, such as symptoms and signs, information on dietary intake and markers of metabolic control, to monitor dietary and medical interventions. The authors summarized recommended indication for CGM monitoring and CGM outcome parameters in Table 6.

**TABLE 6:** Recommended indications for CGM monitoring and CGM outcome parameters for assessment of glucose management in hepatic GSD patients

Settings	Indications
Regular patient care (in‐hospital or at home)	Patient and parent education Repeated hypoglycemia Hypoglycemia unawareness and / or asymptomatic hypoglycemia Clinical GSD evaluation of dietary treatment, in particular but not exclusively when it is difficult to achieve good metabolic control Dietary interventions, such as the introduction of uncooked cornstarch, or changes in the nocturnal dietary treatment Prevention of overtreatment and obesity Initiation of medication effecting glucose homeostasis Monitoring during everyday life at home (such as start of school, physical exercise, psychosocial stress factors, or living independently) Safety reasons
Research setting	Experimental *N*‐of‐1 interventions In addition to the above‐mentioned indications, during novel dietary or medical interventions (ie, gene therapy, mRNA therapy, etc.)
**CGM outcome parameters**	
Descriptive parameters	Mean/median Minimum Maximum Range
Glycemic variation	SD Variance^b^ Coefficient of Variation^b^
Glycemic control^a^	Time under range Level 1 hypoglycemia: ≥ 3.0 and < 3.9 mmol/L Level 2 hypoglycemia: <3.0 mmol/L Level 3 hypoglycemia: a severe event characterized by altered mental and/or physical status requiring assistance^b^ Time in range ≥ 3.9 and ≤ 7.8 mmol/L^b^ ≥ 3.9 and ≤ 10.0 mmol/L Time above range > 7.8 mmol/L^b^ > 10.0 mmol/L
Optional	Cumulative sum analysis^b^ Fourier analysis including frequency, number of frequencies and amplitude of identified frequencies^b^ First and second order derivatives^b^

^a^ As defined according to the American Diabetes Association^15^ 2020.

^b^ Parameters that cannot be directly derived from the Dexcom CLARITY Clinical Portal.

## CONFLICT OF INTEREST

The authors declare no potential conflict of interest.

## AUTHOR CONTRIBUTIONS

All authors substantially contributed to the work and were involved in (a) conception and design of the study and/or analysis and interpretation of data, and (b) revising the article critically for important intellectual content. All authors approved the final manuscript as submitted and agree to be accountable for all aspects of the work. All authors confirm the absence of previous similar or simultaneous publications. Fabian Peeks collected and analyzed the data, wrote the first version of the manuscript, drafted and wrote the final version of the manuscript. Terry G. J. Derks initiated this study, wrote the first version of the manuscript, drafted, and critically reviewed the later versions of the manuscript.

## References

[1] Steunenberg TAH , Peeks F , Hoogeveen IJ , et al. Safety issues associated with dietary management in patients with hepatic glycogen storage disease. Mol Genet Metab. 2018;125:79‐85. 30037503 10.1016/j.ymgme.2018.07.004

[2] Weinstein DA , Steuerwald U , De Souza CFM , Derks TGJ . Inborn errors of metabolism with hypoglycemia: glycogen storage diseases and inherited disorders of gluconeogenesis. Pediatr Clin North Am. 2018;65(2):247‐265. 29502912 10.1016/j.pcl.2017.11.005

[3] Dambska M , Labrador EB , Kuo CL , Weinstein DA . Prevention of complications in glycogen storage disease type Ia with optimization of metabolic control. Pediatr Diabetes. 2017;5:327‐331. 10.1111/pedi.12540 28568353

[4] Kishnani PS , Austin SL , Abdenur JE , et al. Diagnosis and management of glycogen storage disease type I: a practice guideline of the American College of Medical Genetics and Genomics. Genet Med. 2014;16(11):e1. 25356975 10.1038/gim.2014.128

[5] Kishnani PS , Austin SL , Arn P , et al. Glycogen storage disease type III diagnosis and management guidelines. Genet Med. 2010;12(7):446‐463. 20631546 10.1097/GIM.0b013e3181e655b6

[6] Kishnani PS , Goldstein J , Austin SL , et al. Diagnosis and management of glycogen storage diseases type VI and IX: a clinical practice resource of the American College of Medical Genetics and Genomics (ACMG). Genet Med. 2019;21(4):772‐789. 30659246 10.1038/s41436-018-0364-2

[7] Rake JP , Visser G , Labrune P , et al. Guidelines for management of glycogen storage disease type I—European study on glycogen storage disease type I (ESGSD I). Eur J Pediatr. 2002;161(S1):S112‐S119. 12373584 10.1007/s00431-002-1016-7

[8] Sentner CP , Hoogeveen IJ , Weinstein DA , et al. Glycogen storage disease type III: diagnosis, genotype, management, clinical course and outcome. J Inherit Metab Dis. 2016;39(5):697‐704. 27106217 10.1007/s10545-016-9932-2 PMC4987401

[9] Visser G , Rake JP , Labrune P , et al. Consensus guidelines for management of glycogen storage disease type 1b ‐ European study on glycogen storage disease type 1. Eur J Pediatr. 2002;161(Suppl 1):S120‐S123. 12373585 10.1007/s00431-002-1017-6

[10] Heiner‐Fokkema MR , van der Krogt J , de Boer F , et al. The multiple faces of urinary glucose tetrasaccharide as biomarker for patients with hepatic glycogen storage diseases. Genet Med. 2020;22:1915‐1916. 32655139 10.1038/s41436-020-0878-2 PMC7605430

[11] Peeks F , Boonstra WF , de Baere L , et al. Research priorities for liver glycogen storage disease: an international priority setting partnership with the James Lind Alliance. J Inherit Metab Dis. 2020;43(2):279‐289. 31587328 10.1002/jimd.12178 PMC7079148

[12] Vashist S . Continuous glucose monitoring systems: a review. Diagnostics (Basel). 2013;3(4):385‐412. 26824930 10.3390/diagnostics3040385 PMC4665529

[13] Aleppo G , Ruedy KJ , Riddlesworth TD , et al. REPLACE‐BG study group. REPLACE‐BG: a randomized trial comparing continuous glucose monitoring with and without routine blood glucose monitoring in adults with well‐controlled type 1 diabetes. Diabetes Care. 2017a;40(4):538‐545. 28209654 10.2337/dc16-2482 PMC5864100

[14] Aleppo G , Laffel LM , Ahmann AJ , et al. A practical approach to using trend arrows on the Dexcom G5 CGM system for the Management of Adults with diabetes. J Endoc Soc. 2017b;12:1445‐1460. 10.1210/js.2017-00388 PMC5760210 29344577

[15] American Diabetes Association . Standards of medical Care in Diabetes. Diabetes Care Jan. 2020;43:S1‐S2. 10.2337/dc20-Sint 31862741

[16] Danne T , Nimri R , Battelino T . Consensus on use of continuous glucose monitoring. Diabetes Care. 2017;40(12):1631‐1640. 29162583 10.2337/dc17-1600 PMC6467165

[17] Peters AL , Ahmann AJ , Battelino T , et al. Diabetes technology‐continuous subcutaneous insulin infusion therapy and continuous glucose monitoring in adults: an Endocrine Society clinical practice guideline. J Clin Endocrinol Metab. 2016;101(11):3922‐3937. 27588440 10.1210/jc.2016-2534

[18] Rodbard D . Continuous glucose monitoring: a review of recent studies demonstrating improved glycemic outcomes. Diabetes Technol Ther. 2017;19(S3):S25‐S37. 28585879 10.1089/dia.2017.0035 PMC5467105

[19] Polensky WH , Hessler D , Ruedy K , Beck RW . The impact of continuous glucose monitoring on markers of quality of life in adults with type 1 diabetes: further findings from the DIAMOND randomized clinical trial. Diabetes Care. 2017;40:736‐741. 28389582 10.2337/dc17-0133

[20] Vigersky R , Shrivastav M . Role of continuous glucose monitoring for type 2 in diabetes and research. J Diabetes Complicat. 2017;31:280‐287. 10.1016/j.jdiacomp.2016.10.007 27818105

[21] Kaiser N , Gautschi M , Bosanska L . Glycemic control and complications in glycogen storage disease type I: results from the Swiss registry. Mol Gen Metab. 2019;126:355‐361. 10.1016/j.ymgme.2019.02.008 30846352

[22] Kasapkara ÇS , Cinasal Demir G , Hasanoğlu A , Tümer L . Continuous glucose monitoring in children with glycogen storage disease type I. Eur J Clin Nutr. 2014;68:101‐105. 24149443 10.1038/ejcn.2013.186

[23] Maran A , Crepaldi C , Avogaro A , et al. Continuous glucose monitoring in conditions other than diabetes. Diabetes Metab Res Rev. 2004;20:S50‐S55. 15551341 10.1002/dmrr.518

[24] White F , Jones SA . The use of continuous glucose monitoring in the practical management of glycogen storage disorders. J Inherit Metab Dis. 2011;34:631‐642. 21556835 10.1007/s10545-011-9335-3

[25] Herbert M , Pendyal S , Rairikar M , et al. Role of continuous glucose monitoring in the management of glycogen storage disorders. J Inherit Metab Dis. 2018;41:917‐927. 29802555 10.1007/s10545-018-0200-5

[26] Fico G , Hernández L , Cancela J , et al. Exploring the frequency domain of continuous glucose monitoring signals to improve characterization of glucose variability and of diabetic profiles. J Diabetes Sci Technol. 2017;11(4):773‐779. 28627250 10.1177/1932296816685717 PMC5588824

[27] Miller M , Strange P . Use of Fourier models for analysis and interpretation of continuous monitoring glucose profiles. J Diabetes Sci Technol. 2007;1(5):630‐638. 19885131 10.1177/193229680700100506 PMC2769655

[28] Rodbard D . Glucose variability: a review of clinical applications and research developments. Diabetes Technol Ther. 2018;20(S2):5–15. 10.1089/dia.2018.0092 29916742

[29] Agiostratidou G , Anhalt H , Ball D , et al. Standardizing clinically meaningful outcome measures beyond HbA1c for type 1 diabetes: a consensus report of the American Association of Clinical Endocrinologists, the American Association of Diabetes Educators, the American Diabetes Association, the Endocrine Society, JDRF international, the Leona M. and Harry B. Helmsley Charitable Trust, the pediatric Endocrine Society, and the T1D exchange. Diabetes Care. 2017;40(12):1622‐1630. 29162582 10.2337/dc17-1624 PMC5864122

[30] Nano J , Carinci F , Okunade O , et al. A standard set of person‐centred outcomes for diabetes mellitus: results of an international and unified approach. Diabet Med. 2020;37:2009‐2018. 32124488 10.1111/dme.14286

[31] Wortmann SB , Van Hove JLK , Derks TGJ , et al. Treating neutropenia and neutrophil dysfunction in glycogen storage disease type Ib with an SGLT2 inhibitor. Blood. 2020;136(9):1033‐1044. 32294159 10.1182/blood.2019004465 PMC7530374

[32] Peyser TA , Nakamura K , Price D , Bohnett LC , Hirsch IB , Balo A . Hypoglycemic accuracy and improved low glucose alerts of the latest Dexcom G4 platinum continuous glucose monitoring system. Diabetes Technol Ther. 2015;17(8):548‐554. 25961446 10.1089/dia.2014.0415

[33] Wadwa RP , Laffel LM , Shah VN , Garg SK . Accuracy of a factory‐calibrated, real‐time continuous glucose monitoring system during 10 days of use in youth and adults with diabetes. Diabetes Technol Ther. 2018;20(6):395‐402. 10.1089/dia.2018.0150. 29901421 PMC6110124

[34] Dexcom, Inc . Dexcom G4 PLATINUM CGM System with Share. San Diego, CA: Dexcom, Inc.; 2019a. https://www.dexcom.com/dexcom-g4-platinum-share. .

[35] Dexcom, Inc . Make Knowledge your Superpower with the Dexcom G6® CGM System. San Diego, CA: Dexcom, Inc.; 2019b. https://www.dexcom.com/g6-cgm-system. .

[36] Bier DM , Leake RD , Haymond MW . Measurement of “True” glucose production rates in infancy and childhood with 6,6‐dideuteroglucose. Diabetes. 1977;26:1016‐1023. 913891 10.2337/diab.26.11.1016

[37] Huidekoper HH , Ackermans MT , Ruiter AFC , Sauerwein HP , Wijburg FA . Endogenous glucose production from infancy to adulthood: a non‐linear regression model. Arch Dis Child. 2014;99:1098‐1102. 24996789 10.1136/archdischild-2013-305718

[38] Konus OL , Ozdemir A , Akkaya A , et al. Normal liver, spleen, and kidney dimensions in neonates, infants, and children: evaluation with sonography. AJR Am J Roentgenol. 1998;171:1693‐1698. 9843315 10.2214/ajr.171.6.9843315

[39] Sun B , He F , Gao Y , et al. Prognostic impact of visit‐to‐visit glycemic variability on the risks of major adverse cardiovascular outcomes and hypoglycemia in patients with different glycemic control and type 2 diabetes. Endocrine. 2019;64(3):536‐543. 30868413 10.1007/s12020-019-01893-1

[40] Zinman B , Marso SP , Poulter NR , et al. Day‐to‐day fasting glycaemic variability in DEVOTE: associations with severe hypoglycaemia and cardiovascular outcomes (DEVOTE 2). Diabetologia. 2018;61(1):48‐57. 28913575 10.1007/s00125-017-4423-z PMC6002963

[41] El‐Laboudi AH , Godsland IF , Johnston DG , Oliver NS . Measures of glycemic variability in type 1 diabetes and the effect of real‐time continuous glucose monitoring. Diabetes Technol Ther. 2016;18(12):806‐812. 27996321 10.1089/dia.2016.0146

[42] Ajjan R , Slattery D , Wright E . Continous glucose monitoring: a brief review for primary care practitioners. Adv Ther. 2019;36:579‐596. 30659511 10.1007/s12325-019-0870-x PMC6824352

[43] Riddlesworth TD , Beck RW , Gal RL , et al. Optimal sampling duration for continuous glucose monitoring to determine long‐term glycemic control. Diabetes Technol Ther. 2018;20(4):314‐316. 29565197 10.1089/dia.2017.0455

[44] Rousseau‐Nepton I , Huot C , Laforte D , et al. Sleep and quality of life of patients with glycogen storage disease on standard and modified uncooked cornstarch. Mol Genet Metab. 2018;123(3):326‐330. 29223626 10.1016/j.ymgme.2017.09.003

[45] Sechi A , Deroma L , Paci S , et al. Quality of life in adult patients with glycogen storage disease type i: results of a multicenter Italian study. JIMD Rep. 2014;14:47‐53. 24363035 10.1007/8904_2013_283 PMC4213326

[46] Storch E , Keeley M , Merlo L , Jacob M , Correia C , Weinstein D . Psychosocial functioning in youth with glycogen storage disease type I. J Pediatr Psychol. 2008;33(7):728‐738. 18296725 10.1093/jpepsy/jsn017

[47] Klonoff DC , Ahn D , Drincic A . Continuous glucose monitoring: a review of the technology and clinical use. Diabetes Res Clin. 2017;133:178‐192. 10.1016/j.diabres.2017.08.005 28965029

[48] Woldaregay AZ , Årsand E , Botsis T , Albers D , Mamykina L , Hartvigsen G . Data‐driven blood glucose pattern classification and anomalies detection: machine‐learning applications in type 1 diabetes. J Med Internet Res. 2019;21(5):e11030. 31042157 10.2196/11030 PMC6658321

[49] Kriventsov S , Lindsey A , Hayeri A . The Diabits app for smartphone‐assisted predictive monitoring of glycemia in patients with diabetes: retrospective observational study. JMIR Diabetes. 2020;5(3):e18660. 32960180 10.2196/18660 PMC7539161

[50] Martini PGV , Guey LT . A new era for rare genetic diseases: messenger RNA therapy. Hum Gene Ther. 2019;10:1180‐1189. 10.1089/hum.2019.090 31179759

[51] Olafsdottir AF , Polonsky W , Bolinder J . A randomized clinical trial of the effect of continuous glucose monitoring on nocturnal hypoglycemia, daytime hypoglycemia, glycemic variability, and hypoglycemia confidence in persons with type 1 diabetes treated with multiple daily insulin injections (GOLD‐3). Diabetes Technol Ther. 2018;20(4):274–284. 29608107 10.1089/dia.2017.0363 PMC5910048

[52] Peeks F , Steunenberg TAH , de Boer F . Clinical and biochemical heterogeneity between patients with glycogen storage disease type Ia: the added value of CUSUM for metabolic control. 2017;40(5):695–702. 10.1007/s10545-017-0039-1 PMC5579135 28397058

